# Synthesis and Characterization of Fucoidan-Chitosan Nanoparticles Targeting P-Selectin for Effective Atherosclerosis Therapy

**DOI:** 10.1155/2022/8006642

**Published:** 2022-09-09

**Authors:** Mingying Liu, Yu Zhang, Xuewei Ma, Bo Zhang, Yinghui Huang, Jinghong Zhao, Shaobo Wang, Yan Li, Yingguo Zhu, Jiachuan Xiong, Ting He, Yue Wang, Wenhao Han, Ke Yang, Xianjin Bi, Yong Liu, Hao Zhang

**Affiliations:** ^1^School of Comprehensive Health Management, Xihua University, Chengdu, Sichuan 610039, China; ^2^National Innovation and Attracting Talents “111” Base, Key Laboratory of Biorheological Science and Technology, Ministry of Education, College of Bioengineering, Chongqing University, Chongqing 400030, China; ^3^Department of Nephrology, The Key Laboratory for the Prevention and Treatment of Chronic Kidney Disease of Chongqing, Kidney Center of PLA, Xinqiao Hospital, Army Medical University (Third Military Medical University), Chongqing 400037, China

## Abstract

Atherosclerosis is the key pathogenesis of cardiovascular diseases; oxidative stress, which is induced by the generated excess reactive oxygen species (ROS), has been a crucial mechanism underlying this pathology. Nanoparticles (NPs) represent a novel strategy for the development of potential therapies against atherosclerosis, and multifunctional NPs possessing antioxidative capacities hold promise for amelioration of vascular injury caused by ROS and for evading off-target effects; materials that are currently used for NP synthesis often serve as vehicles that do not possess intrinsic biological activities; however, they may affect the surrounding healthy environment due to decomposition of products. Herein, we used nontoxic fucoidan, a sulfated polysaccharide derived from a marine organism, to develop chitosan–fucoidan nanoparticles (CFNs). Then, by binding to P-selectin, an inflammatory adhesion exhibited molecule expression on the endothelial cells and activated platelets, blocking leukocyte recruitment and rolling on platelets and endothelium. CFNs exhibit antioxidant and anti-inflammatory properties. Nevertheless, by now, the application of CFNs for the target delivery regarding therapeutics specific to atherosclerotic plaques is not well investigated. The produced CFNs were physicochemically characterized using transmission electron microscopy (TEM), together with Fourier transform infrared spectroscopy (FTIR). Evaluations of the *in vitro* antioxidant as well as anti-inflammatory activities exhibited by CFNs were based on the measurement of their ROS scavenging abilities and investigating inflammatory mediator levels. The *in vivo* pharmacokinetics and binding efficiency of the CFNs to atherosclerotic plaques were also evaluated. The therapeutic effects indicated that CFNs effectively suppressed local oxidative stress and inflammation by targeting P-selectin in atheromatous plaques and thereby preventing the progression of atherosclerosis.

## 1. Introduction

Atherosclerosis, featuring inflammatory cells in the walls of medium and large-sized arteries as well as the accumulation of lipids, is a major cause of death over the world [1, 2]. The pathogenesis of atherosclerosis involves the expression of macrophages that contain proinflammatory signaling pathways and excess oxidized lipids. Reactive oxygen species (ROS) remarkably affect protein modification, cell apoptosis, breakage of DNA strands, and alteration in the oxidation of low-density lipoprotein (LDL)-cholesterol that is believed to promote foam cell formation and induce endothelial dysfunction as well as increase the adhesion molecules' expression and scavenger receptors [3]. Antioxidants and anti-inflammatory agents represent some of the current available treatment modalities; however, many of these therapies may exhibit some limitations due to their nonspecific distribution and short retention time in atherosclerotic plaques [4]. Therefore, alternative therapeutic strategies must be developed.

As most atherogenesis processes occur at the nanoscale, the use of nanoparticles (NPs) is a promising approach for targeted therapy of atherosclerosis by virtue of their fine-tunable properties, size, and their capability of loading different therapeutics such as proteins, nucleic acids, and drugs [5]. The application of nanotechnologies for atherosclerosis and cardiovascular disease has been broadly investigated. For example, when NPs target a specific injury site within the heart, controlled therapeutic molecules releasing can be adsorbed or conjugated [6]. Therefore, NP-based targeting strategies appear to be effective for molecular imaging and for atherosclerosis treatment therapies. Typically, there are two types of interventions that guide the choice of material for antioxidative NP treatment of atherosclerosis, and these are (1) drug delivery and (2) plaque visualization with its processes [7]. Thus, numerous majority of polymers, capable of being integrated into specific drug carries to achieve efficient delivery [8] (for example, liposomes), have been successfully applied in atherosclerosis drug delivery due to their lipid-like properties and ability to manipulate target sites by altering bilayer constituents [9]. Polyester materials such as poly(L-lysine) (PLL), polyethylenimine (PEI), and poly(lactic-co-glycolic acid) (PLGA) are some other commonly used polymers in this regard as they are capable of delivering antioxidants or other polar cargo such as proteins [10]. However, while these materials are vehicles for targeted delivery to the atherosclerotic plaques, they can also affect the surrounding healthy environment or may induce inflammatory responses due to their hydrolytic decomposition products [11]. Thus, due to their native tissue composition, natural substances can serve as a proper alternative approach in the development of novel NPs due to their native tissue compositions.

The potential of natural biomaterial compounds exhibiting pharmacological properties for cancer therapies has been demonstrated in numerous studies [12, 13]. However, developing NPs using these functional materials for the treatment of atherosclerosis can be a challenge due to imprecise targeting sites [6]. P-selectin is an inflammatory adhesion molecule mediating the rolling of hemocytes on the endothelium surface as well as initiating leukocytes attachment that circulate within the blood to endothelial cells, platelets, and other white blood cells at inflammation or tissue injury sites [14]. The crucial role of P-selectin in vascular disease progression has been confirmed in ApoE^−/−^ mouse crossed with P-selectin knockout models that exhibit a significant reduction in leukocyte recruitment in atherosclerosis plaque. Therefore, P-selectin acts as an attractive potential therapeutic target in the context of cardiovascular disease, including atherosclerosis, as it is capable of initiating cell activation and adhering to platelets and endothelial cells [15]. A previous study indicated that sulfated polysaccharides and sulfated oligosaccharides such as fucoidan, dextran sulfate, and heparin could achieve effective targeting of P-selectin positively related to metastatic growth [16]. Among these, fucoidan is a sulfated polysaccharide derived from marine organisms and displays several advantageous biological behaviors, making it an excellent candidate for NP development with regard to atherosclerosis treatment; fucoidan exhibits antioxidative [17], anticoagulant [18], and anti-inflammatory activities [19] that are known to inhibit hydroxyl radical and superoxide radical formations [20]. Reports demonstrated that fucoidan alleviates cytotoxicity by decreasing oxidative stress *in vivo* [21]. The structure of fucoidan includes an *α*(1-3)-L-fucose linear backbone with sulfate substitution that allows it to bind to P-selectin with high affinity and exert antagonism of selective function in myocardial ischemia-perfusion injury models in rats and pigs [22–25]. Therefore, the development of NPs based on fucoidan and selective P-selectin is an important direction for atherosclerosis therapy.

**Scheme 1:**
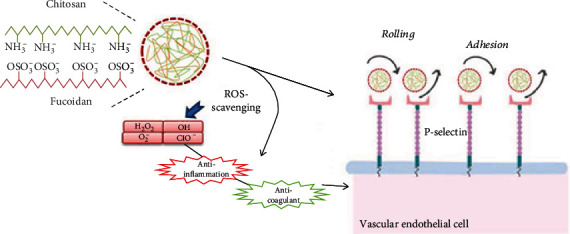
Illustration of synthesizing CFNs with P-selectin targeting potential to block leukocyte recruitment and rolling on platelets and endothelium in atherosclerotic plaques. The effects of CFNs were recognized by attenuating systemic oxidative stress as well as inflammatory cell infiltration in plaques.

In this study, a novel P-selectin-targeting drug delivery system was established by using nontoxic fucoidan. The nanoplatform was constructed based on the polyelectrolyte interactions of chitosan (CS), a chitin-derived cationic polysaccharide that is and was broadly applied as a carrier for improving as well as controlling drug release [26]. In addition to their biological behaviors, fucoidans have also used to stabilize the NPs and to study the behavior of their aqueous suspension [27]. On this basis, the developed fucoidan–chitosan nanoparticle (CFN) was used as a nanoplatform and to P-selectin and block leukocyte recruitment and rolling on platelets and the endothelium (Scheme 1). To our knowledge, literature about the P-selectin-targeting NPs development with intrinsic antioxidative activities for targeted therapy of atherosclerosis has not been reported. The prepared CFNs were physicochemically characterized by using transmission electron microscopy (TEM), together with Fourier transform infrared spectroscopy (FTIR). The evaluation on *in vitro* anti-inflammatory and antioxidant activities of CFNs was based on the measurement of their ROS-scavenging abilities and the investigation of the inflammatory mediator levels like IL-6 and NO in lipopolysaccharide (LPS)-stimulated macrophages. The *in vivo* pharmacokinetics and binding efficiency of CFNs to atherosclerotic plaques were evaluated using the cardiovascular disease-related suppressor protein klotho as a positive control. Moreover, the therapeutic effects of CFNs were assessed, and the results indicated they could effectively suppress local oxidative stress and inflammation by targeting inflammatory cells and atheromatous plaques, to thereby prevent the progression of atherosclerosis.

## 2. Experimental Section

### 2.1. Materials

Fucoidan (from *Fucus vesiculosus*), chitosan (CS: deacetylation degree ≥75%), nitro blue tetrazolium (NBT), nicotinamide adenine dinucleotide-reduced (NADH), phenazine methosulfate (PMS), 1,1-diphenyl-2-picrylhydrazyl (DPPH), Oil Red O (ORO), LPS, hydrogen peroxide (H_2_O_2_), and fluorescein isothiocyanate (FITC) were provided by the Sigma Chemicals Co. (St. Louis, MO, USA). Penicillin, fetal bovine serum (FBS), streptomycin, and Dulbecco's modified Eagle medium (DMEM) were purchased from Gibco (USA). Additionally, 3,3′-dioctadecyloxacarbocyanine perchlorate (DiO), 4′,6-Diamidino-2- phenylindole (DAPI) and anhydrous dimethyl sulfoxide (DMSO), and recombinant murine interferon-*γ* (INF-*γ*) were bought from Beyotime (China). Human highly oxidized low-density lipoprotein (oxLDL) was bought from Yiyuan Biotechnologies (China).

### 2.2. Preparation of CFNs

CFNs were prepared using a polycation/polyanion self-assembly approaches by ultrasonication at room temperature. Various concentrations of fucoidan (ranging from 0.75 to1.5 mg/mL) were studied to optimize different parameters for CFNs size and polydispersity index (PDI). CFNs possess a theoretical CS-to-fucoidan ratio of 3 : 1, and the concentrations of chitosan were various (ranging from 2.25 to 4.5 mg/mL) according to those of fucoidan. CFNs were prepared by adding 3 mL of fucoidan solution (pH 9) to 3 mL of CS/acetic acid solution (pH 5) through probe-type ultrasonicating (pulse-off 7 s and pulse-on 3 s; total 30 s) in an ice bath. Immediately after CFN was produced, 120 mM NHS+300 mM EDC were added to the CFNs solution and stirred at 600 rpm for 2 h. Then, CFNs were collected by 300 kDa vivaspin filters centrifugation at 4000 rpm for 15 min. Ultrapure water was used to wash CFNs twice, and the supernatants were removed and resuspended for further study. All centrifugation steps were added with glucose for avoiding NP aggregation, and CFNs were filtered with 0.22-*μ*m filters [28–30].

### 2.3. Characterization of CFNs

Fourier transform infrared spectroscopy (FTIR; Bruker Tensor 27) assisted in analyzing the peak variation in CFNs. Measurement of size distribution, polydispersity index (PDI), and zeta potential of CFNs was performed with a Zetasizer Nano ZS (Malvern Instruments Ltd., Worcestershire, UK) at 25°C. Dynamic light scattering (DLS) assisted in characterizing the CFNs. The CFNs morphology was observed using TEM (JEOL, Japan), and a drop of CFNs suspension was applied to a 200-mesh copper grid for about 10 min, then the surface water was remove by taping the filter paper onto the grid, and the NPs were fixed for 2–3 h in 1% formaldehyde. Then by using an alkaline bismuth solution, the samples were positively stained [26].

### 2.4. Antioxidant Activity Assays

#### 2.4.1. DPPH Scavenging Activity

The scavenging activity regarding DPPH free radicals was assessed using a previously established protocol with slight modifications [31]. Briefly, 2 mL of CFN sample solutions at varying concentrations (0.1–5 mg/mL) were added to 2 mL of a 0.2 mM DPPH in ethanol solution, and the reaction mixture underwent vigorous shaking, followed by 30 min of incubation in the dark at room temperature. Measurement of absorbance of the resulting solution was performed at a wavelength of 517 nm. We used vitamin C as a positive control [32], and the calculation of the scavenging effect was based on the equation below:

(1) $$\begin{array}{c} \text{Scavenging effect }\left(\%\right)=\left(1-\frac{\text{CS or Fu value}}{\text{Control}}\right)\times 100\%. \end{array}$$

#### 2.4.2. Superoxide Radical Scavenging Assay

Superoxide radicals were produced in the PMS–NADH system that contained 3 mL of Tris–HCl buffer (16 mM, pH 8.0), 338 *μ*M NADH, 72 *μ*M NBT, 30 *μ*M PMS, and various concentrations of CFN samples (0.01–0.2 mg/mL). The earlier prepared mixture received 5 minutes of incubation at room temperature, and the absorbance at 560 nm against a blank was recorded [33]. For control group, Tris–HCl buffer was used as a substitute. The calculation of scavenging effect on superoxide radical was based on the equation below:

(2) $$\begin{array}{c} \text{Scavenging effect }\left(\%\right)=1-\frac{\text{Asample}560\text{ nm}}{\text{Acontrol }560\text{ nm}}\times 100\%. \end{array}$$

#### 2.4.3. Hydroxyl Radical Scavenging Activity

The scavenging activity of the CFNs against the hydroxyl radicals was investigated according to a previous protocol [20]. The reaction mixture that contained different samples (0.14–3.46 mg/mL) received 30 min of incubation by 0.5 mL of EDTA-Fe (2 mM), 1 mL of H2O2 (3%), and 360 *μ*g/mL of crocus in 4.5 mL of sodium phosphate buffer (150 mM, pH 7.4) at 37°C, and absorbance at 520 nm was monitored for detecting the presence of the hydroxyl radical. In the control, sodium phosphate buffer was used to replace H_2_O_2_, and the distilled water was used as the substitute of the sample. The scavenging effect on the hydroxyl radicals was based on the equation below:

(3) $$\begin{array}{c} \text{Scavenging effect }\left(\%\right)=1-\frac{\text{Asample}520\text{ nm}}{\text{Acontrol }520\text{ nm}}\times 100\%. \end{array}$$

### 2.5. Intracellular ROS Generation in Macrophages

The cultured RAW 264.7 cells in 12-wellplates (2.0 × 10^5^ cells/well) were allowed to attach for 24 h before treatment. Then the cells underwent 24 h of treatment by using various doses of CFNs and 100 ng/mL of LPS and of 100 IU/mL IFN-*γ*. Fresh medium was served for treating the normal group, while the model group underwent 24 h stimulation by using LPS/IFN-*γ* without CFNs. The medium was then changed to a serum-free DMEM that contained 20 *μ*M of DCFH-DA, together with 30 minutes of incubation at 37°C. Subsequently, an inverted fluorescence microscope (Olympus IX71, Tokyo, Japan) helped to observe cells containing the fluorescent compound dichlorofluorescein (DCF). Next, the cells were washed in phosphate-buffered saline (PBS) once, collected by 0.25% trypsinization, rewashed, and finally resuspended in PBS. The flow cytometry (Accuri C6, BD Biosciences) assisted in intracellular fluorescent signal measurement.

### 2.6. Proinflammatory Cytokine Levels Measurement in RAW 264.7 Macrophage Cultures by Enzyme-Linked Immunosorbent Assay (ELISA)

The RAW 264.7 macrophages (3 × 10^4^ cells/well) were seeded into a 24-well plate, followed by 24 h of preincubated with 100 ng/mL of LPS, 100 IU/mL of IFN-*γ*, and the indicated concentrations of CFNs. After that, an ELISA (Wuhan Boster Biological Engineering Co. Ltd., Wuhan, China) assisted in measuring the proinflammatory cytokine (IL-6, IL-1*β*, and TNF-*α*) levels by collecting 100 *μ*L of culture supernatants from each individual treatment. For positive control, LPS was used to stimulate proinflammatory cytokines and to validate the ELISA protocol.

### 2.7. Nitric Oxide Assay

RAW 264.7 Cells (1.0 × 10^5^ cells/well) were plated into 24-well plates and preincubated with the indicated concentrations of CFNs, 100 ng/mL of LPS with 100 IU/mL of IFN-*γ* for 24 h. After that, the cell culture supernatants were collected, and an available commercial test kit (Nanjing Jiancheng Bioengineering Institute, China) assisted in quantifying the NO generation. Then calculation on the NO-inhibiting capacity exhibited by CFNs was performed.

### 2.8. Effect of CFNs Treatment on Foam Cell Formation

The RAW 264.7 macrophages (3 × 10^4^ cells/well) were seeded into a 24-well plate, followed by 24 h of preincubation by 100 ng/mL of LPS, 100 IU/mL of IFN-*γ*, and the indicated doses of CFNs for 24 h. Cells then underwent 48 h of incubation by 50 *μ*g/mL of oxLDL. The fresh medium served for normal control group, while the model group only received stimulation by oxLDL. Cells were washed by 0.5 M HCl in 70% ethanol, fixed by 10% neutral buffered formalin, and then stained by 0.3% ORO and hematoxylin. The optical microscopy served for cell observation.

### 2.9. *In Vitro* Cytotoxicity Evaluation

*In vitro* cell cytotoxicity was determined using a counting kit-8 assay. Mouse RAW 264.7 macrophage and endothelial cells (1.0 × 10^4^ cells/well) were incubated in triplicate in 96-well plates, together with 24 h of attachment before being exposed different concentrations (0.75-2 mg/mL) of CFN solutions for 48 and 72 h. Then, each well was added with WST-8 for additional 4 h of incubation; consequently, an automatic microplate reader assisted in determining the absorbance values at 450 nm. Normalization of values was performed to the values of untreated control samples [26, 34–36].

### 2.10. Apoptosis Detection

RAW 264.7 macrophages (1 × 10^6^ cells/mL) were pretreated in 6-well plates at in fresh medium that contained different concentrations of CFNs. After 2 h, the cells received 24 h of incubation by 500 *μ*M H_2_O_2_ in fresh medium, and we used medium for culturing the cells in the normal control group alone. The collected cells were then underwent 15 minutes of staining by a FITC Annexin V apoptosis detection kit with propidium iodide (Beyotime Biotechnology, China) in dark at 4°C. Flow cytometry assisted in apoptosis assay [34, 37].

### 2.11. Observation of Cellular Uptake of NPs via Macrophages

For preparing the fluorescein isothiocyanate (FITC)-tagged NPs, FITC was completely dissolved in DMSO (0.2 mg/mL). Then, at ambient temperature 200 *μ*L of the FITC solution was added to 10 mL of CS (1% in 0.1 M CH3COOH) in the dark. The CFNs were then prepared using a similar protocol to that described above [38].

FITC-labeled CFNs were used to investigate the uptake of these NPs by macrophages cells [36]. RAW 264.7macrophage cells were cultured in 12-well plates (2 × 10^5^ cells/well) for 12 h. Then they were exposed to 1.5 mg/mL of FITC-tagged CFNs, 100 ng/mL of LPS, and 100 IU/mL of IFN-*γ* in serum-free medium; at predetermined points, the cells were stained with DiI (red, Beyotime Biotechnology, China) and fixed with 4% (w/v) paraformaldehyde for nuclear staining with DAPI (blue). Cells were imaged using fluorescence microscopy (OLYMPUS BX51) at a continual exposure time [34, 35].

### 2.12. Mouse Vascular Endothelial Cells (MVECs) Uptake of CFNs and Impact of P-Selectin Inhibitor

MVECs underwent 24 h of culturing in 12-well plates (3 × 10^5^ cells/well), followed by the incubation with 1.5 mg/mL of FITC tagged CFNs. At predetermined time, the cells were stained with DiI (red) and fixed with 4% (w/v) paraformaldehyde for nuclear staining with DAPI (blue). A confocal laser scanning microscope (CLSM, Mannheim, Germany) assisted in cell imaging.

The effect of P-selectin inhibitor on the CFNs uptake was evaluated using flow cytometry. MVECs received 24 h of culturing in 12-well plates (3 × 10^5^ cells/well), and then the inhibitor of P-selectin (KF 38789, Tocris) was added to the cells at a dose of 0.5-2 *μ*M. Following 1 h of pretreatment, MVECs underwent 6 h of incubation by 1.5 mg/mL of FITC-tagged CFNs. The cells were stained with DiI (red) and fixed using 4% (w/v) paraformaldehyde for nuclear staining with DAPI (blue). They were then washed three times with PBS, and after trypsinized, they were resuspended in PBS, and flow cytometry (Accuri C6, BD Biosciences) assisted in the fluoresce intensity analysis [16].

### 2.13. P-Selectin Protein Analysis by Western Blotting

MVECs underwent 24 h of culturing in 12-well plates (3 × 10^5^ cells/well), followed by the incubation with 1.5 mg/mL of CFNs. After predetermined time periods, analysis buffer (10 mM Tris-HCl, pH 7.5), 1% Triton X-100 (Solarbio, China), 50 mM NaCl, 20% glycerol, 1 mM EDTA, and 1 mM PMSF were used for extracting cell lysates. Proteins extracting from the cell lysates were separated by 8% SDS-PAGE, transferred to a polyvinylidene fluoride (PVDF) membrane (Roche, Germany), and blotted with PL-1 antibody. Bound antibodies were detected using an HRP-conjugated secondary antibody. The membrane received the incubation in Tris-buffered saline that contained 3% bovine serum albumin (BSA) with 0.1% Tween-20 (TBST) prior to incubation in rabbit P-selectin (CD62P) antibody (PA5-79973, Thermo Fisher Scientific) or rabbit anti-GAPDH antibody (ab181602, Abcam, Inc., MA) overnight at 4°C. An imaging system (Bio-Rad, USA) was used to visualize the protein bands [39, 40].

### 2.14. Animals

The experiments and animal care were performed according to the National Institutes of Health guidelines. The Army Medical University Ethics Committee specifically approved this study. Male apolipoprotein E-deficient (ApoE^−/−^) mice (approximately 8 weeks old) were provided by the Peking University Health Science Center (China). 7 days of acclimatization later, the mice were received various experiments.

### 2.15. Atherosclerotic Plaques Targeting by CFNs in ApoE^−/−^ Mice

Male ApoE^−/−^ mice received a western-type diet (0.5% sodium cholate, 1.25% cholesterol, and 15% fat) for 3 months for developing atherosclerosis. FITC-tagged CFNs were intravenously administered at a concentration of 100 mg/kg body weight. Mice were sacrificed and perfused according to the aforementioned procedure with 0.9% saline and 4% paraformaldehyde at 6 and 12 h postinjection, with the aortas and main organs including the kidney, liver, heart, lung, and spleen being harvested, IVIS spectrum imaging system assisted in capturing the *ex vivo* images, and the Living Image 4.5 software served for the MFI analysis. To examine the aortic plaques distribution of CFNs, aortic samples were embedded and a series of 8*-μ*m-thick frozen sections were obtained in optimal cutting temperature compound. After 20 min of fixation with precooled acetone at room temperature, the permeabilized slides underwent 1 h of blocking by 0.1% saponin in PBS, 1% Triton X-100, 4% BSA, and 10% normal goat serum (NGS). Then the slides underwent one night of incubation at 4°C by 5 *μ*L of rabbit P-selectin (CD62P) antibody, and this was followed by 1 h incubation using Cy3-labeled goat antirat IgG (H + L) (1: 500, Beyotime, China) and DAPI (Beyotime, China). CLSM served for observing the slides [34, 41].

### 2.16. Treatment Protocol for Atherosclerotic Mice

ApoE^−/−^ mice received a western-type diet for 12 weeks. After 4 weeks, we randomly divided mice into three groups (*n* = 8) which then received various treatments for an additional 8 weeks. The klotho (positive control) and CFNs groups were intravenously injected with free 20 *μ*g/kg of klotho and 100 mg/kg of CFNs (CS: Fu 3 : 1, Fu concentration: 1.5 mg/mL). We treated mice in the model control group by using saline, dissolved both klotho and CFNs in saline, and conducted intravenous injection on all formulations were i.v. injected twice per week [42, 43].

### 2.17. Histology and Immunofluorescence Analysis

After the various treatments, ApoE^−/−^ mice were euthanized. The pathological change degree in the lesion area of the aorta from the heart to the iliac bifurcation was evaluated. In brief, the fixed aorta was opened longitudinally, and plaque area was quantified by the ORO staining. To determine the extent of atherosclerosis at the aortic roots, we placed the roots in an optimal cutting temperature compound followed by the quick freezing. The tissues were serially cross-sectioned at 5 *μ*m intervals, followed by receiving ORO, hematoxylin–eosin (H&E), and Masson's trichrome staining. The sections were stained with an anti-CD68 antibody for evaluating the macrophages. An ELISA kit (Beyotime Biotechnology, China) was employed for confirming the serum levels regarding TNF-*α* and IL-1*β* in the collected blood samples [41].

### 2.18. Vascular Superoxide Anion Generation Detection Using Dihydroethidium Staining

The fluorescent dye dihydroethidium (DHE) (Beyotime Biotechnology., China) assisted in measuring the vascular ROS formation (primarily superoxide). We placed brachiocephalic artery samples in Tissue-Tek O.C.T. compound and prepared 8-*μ*m sections. This was followed by 10 min of incubation using 2% Triton X-100 at 21°C. Subsequently, blocking was performed by using 5% BSA in PBS; next, a 2 *μ*M DHE in Krebs solution was applied to each slide and evaluated by fluorescence microscopy, and the Image-Pro Plus 6.0 software served for the fluorescence intensity analysis [34, 44, 45].

### 2.19. PLT Isolation and the P-Selectin Expression in Peripheral Blood

After 2 months of treatment, the mice were sacrificed, with their blood collected in EDTA spray-coated tubes. This was followed by 12 min of centrifugation at 2500 rpm at room temperature. The harvested supernatants centrifuged for 30 min at 3200 rpm at 4°C and then extracted, and PLTs were collected. Next, the expression of P-selectin in peripheral blood was evaluated. We suspended the PLTs in 50 *μ*L of PBS (0.1 M), followed by adding 5 *μ*L of rabbit P-selectin (CD62P) antibody. The PLTs underwent 1 h of incubation at 37°C, following which PBS (0.1 M) was employed for washing them twice and the cleaned PLTs underwent centrifugation at 3000 rpm; this was followed by 1-h incubation with FITC-labeled goat antirabbit IgG (H + L) (1: 500, Beyotime, China). A Cell Lab Quanta SC Coulter flow cytometer (Beckman Coulter, USA) was adopted for measuring the PLTs [46].

### 2.20. Long-Term Safety of CFNs

Typical hematological parameters, namely red blood cell count (RBC), white blood cell count (WBC), and biochemical markers relevant to the functions of the liver, kidney, and serum, were measured, including blood urea nitrogen (BUN), aspartate aminotransferase (AST), alanine aminotransferase (ALT), total cholesterol (TC), serum creatinine (SCr), LDL, triglyceride (TG), and high-density lipoprotein (HDL). We collected different organs (lung, liver, heart, kidneys, and spleen) and fixed them in 4% paraformaldehyde (overnight). Then they were sectioned at 4 *μ*m, followed by the H&E staining for histological analysis [47].

### 2.21. Statistical Analysis

Data are in the form of the mean ± standard deviation (SD). SPSS 20.0 was employed to perform statistical analysis, and a one-way ANOVA test assisted in the statistical analysis specific to experiments that is involving over two groups. Comparisons between any two groups were performed using two-tailed unpaired Student's *t*-test. Statistical significance was set at *P* < 0.05.

## 3. Results

### 3.1. Characterization of CFNs

#### 3.1.1. FT-IR Analysis of the CFNs

**Figure 1:**
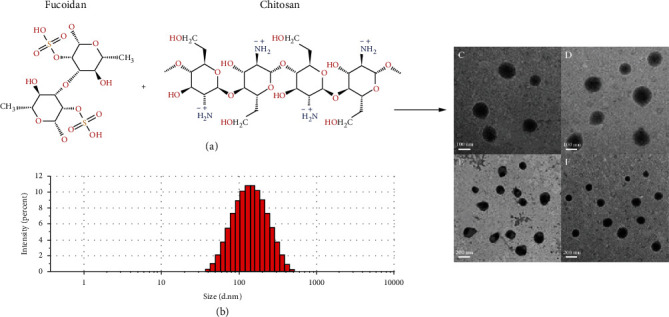
(a) Synthesis schemes of P-selectin-targeted CFNs. (b) Size distribution profile of CNFs. (c–f) Representative transmission electron microscopy (TEM) image of CNFs. Scale bars: (c and d) 100 nm; (e and f) 200 nm.

**Figure 2:**
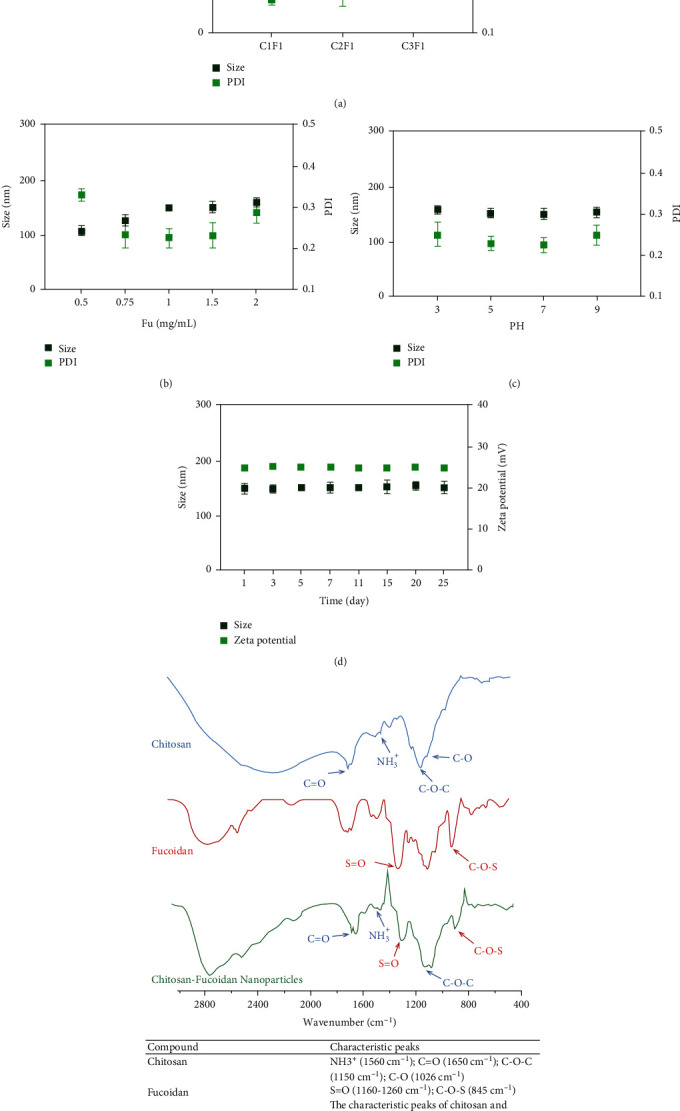
Characterization of CNFs. (a) CFNs' size and PDI at different CH:Fu ratio (Fu concentrations: 1 mg/mL). (b) CFNs' size and PDI at different fucoidan concentrations. Ratio of CH:Fu was set at 3 : 1; the concentrations of chitosan were various according to fucoidan. (c) Influence of pH over CNFs' size and PDI (Fu concentrations: 1.5 mg/mL, CH:Fu 3 : 1). (d) Storage stability of CFNs (Fu concentrations: 1.5 mg/mL, CH:Fu 3 : 1): size and zeta potential at 4°C, pH:7.0. (e) The FTIR spectra of CS, fucoidan, and CFNs. Data in (a)–(d) are mean ± S.D. (*n* = 3).

The electrostatic interaction of fucoidan and chitosan is illustrated in Scheme 1 and Figure 1(a); Figure 2 presents the FT-IR spectra regarding chitosan, fucoidan, and CFNs. Chitosan exhibits characteristic peaks near 1560 and 1650 cm^−1^ that are associated with the NH^3+^ (protonated amino group) bending vibrations and the C=O (carbonyl group) stretching of the secondary amide, respectively. Furthermore, in the CS spectrum, C–O–C symmetric stretching occurred at 1150 cm^−1^ and C–O skeletal vibration occurred at 1026 cm^−1^. In the FT-IR spectrum regarding fucoidan, the absorption bands near 1160-1260 cm^−1^ corresponded to the S=O asymmetric stretching regarding the sulfate group and that at 845 cm^−1^ corresponded to the C-O-S stretching [24]. The spectrum regarding the CFNs covers all the characteristic absorptions regarding CS and fucoidan, indicating the noncovalent interactions between CS and fucoidan. The amino group with positive charge on CS and sulfate group with negative charge on fucoidan formed CFNs through electrostatic interactions [22] (Figure 2(e)).

#### 3.1.2. NP Characterization

To optimize the production parameters of CFNs over size and PDI, several polymer concentrations and ratios were first tested in this study, and the results indicated that an increase in chitosan:fucoidan ratio led to the formation of smaller CFNs (Figure 2(a)). For a chitosan:fucoidan ratio of 3 : 1, the CFNs presented a slightly higher PDI than that obtained using a 2 : 1 ratio. Therefore, the 3 : 1 ratio was used in further experiments. The results displayed a similar formation size of CFNs with varying polymer concentrations. However, for both high and low polymer concentrations at 3 : 1 (CH:Fu), the PDI of the obtained CFNs were increased, particularly when the fucoidan concentrations were <0.75 mg/mL (PDI>0.3), thereby indicating instability of the CFNs (Figure 2(b)). Therefore, we selected fucoidan concentrations of fucoidan in the range of 0.75-1.5 mg/mL for further experiments.

Size variability is one of the advantages of NPs for the treatment of atherosclerosis, as NPs can persist in an atherosclerotic plaque microenvironment that is also characterized by a vascular leakage. As the gap junction size between the endothelial cells may range from 100 to 600 nm [48], to achieve increased intraplaque accumulation and distribution for improving therapeutic response, the size of the CFNs should be <600 nm. In this study, the CFNs sizes were approximately 150.0 ± 9.4 nm when the pH was 7.0 (Figure 2(c)), which was the normal value of human body under physiological conditions. Similarly CFNs have a size of 152 nm, PDI = 0.192, and zeta potential of 25 mV by DLS (Table 1), thereby indicating that the stability of CFNs would guarantee the maintenance of their function *in vivo.* Meanwhile, CFNs with a positive charge are favorable, relying on the interaction of NPs with cell populations along the vascular wall that contains cell membranes with negative charge, such as endothelial cells, platelets, and vascular smooth muscle cells (VSMC) [49]. Additionally, for the deposition of particles with a diameter<500 nm, Brownian diffusion is also a potential mechanism [50].  Figure 2(d) shows the storage stability of the CFNs. Under physiological conditions, the CFNs maintain compactness with slight particle size variation over 25 days; the particles also maintain a positive zeta potential when pH value is 7.0. The CFN morphology in the dry state was also observed using TEM, CFNs are essentially spherical in shape (Figures 1(c)–1(f)), and the diameter is approximately 130-200 nm (Figure 1(b)).

### 3.2. Antioxidant Activity Assays

#### 3.2.1. Scavenging Activity against DPPH Radicals

**Figure 3:**
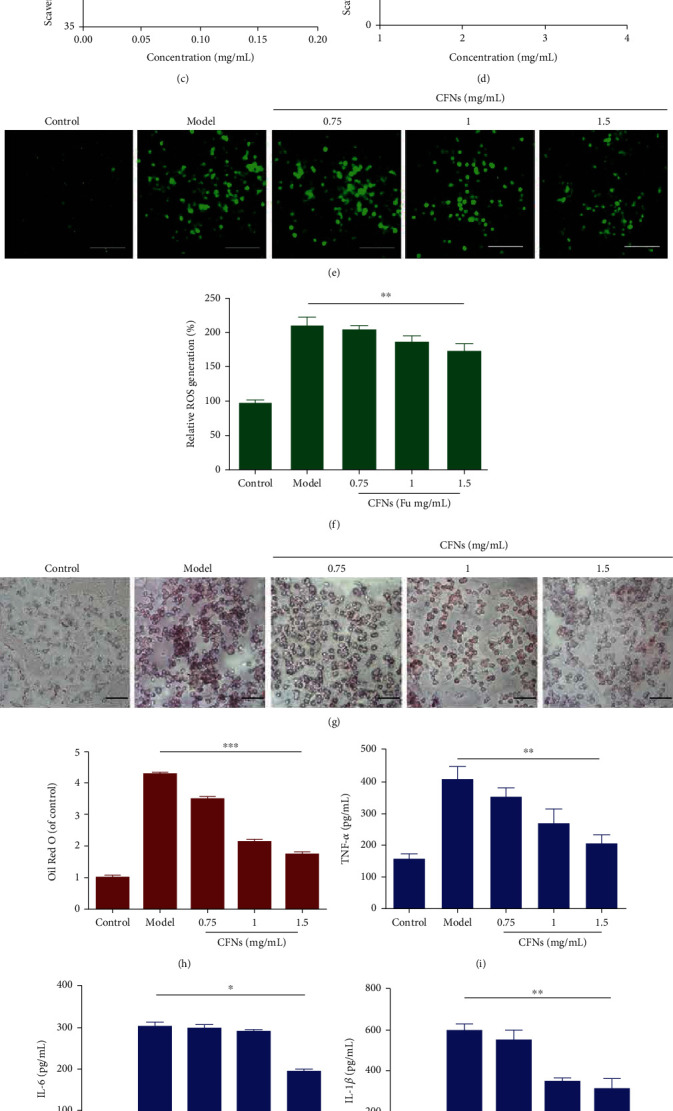
(a) DPPH radical scavenging activity of CNFs (CH:Fu 3 : 1). (b) DPPH radical scavenging effect of chitosan, fucoidan, and vitamin C. (c) Superoxide radicals and (d) hydroxyl radicals scavenging activity of CNFs (CH:Fu 3 : 1). (e) Fluorescence images and (f) quantification by flow cytometry of ROS generation of RAW264.7 cells. (g) Oil Red O (ORO) staining images and (h) quantified contents of oxLDL-induced foam cell formation in macrophages. (g–j) Effect of CFNs on typical inflammatory cytokines TNF-*α* (i), IL-6 (j), IL-1*β* (k), and NO (l) produced by LPS/IFN-*γ*-induced RAW264.7. Scale bars: (e and g) 100 *μ*m. Data are means ± S.D. (*n* = 3).

As a stable free radical, DPPH can be reduced to DPPH-H when encountering a proton-donating substance, and the process can indicate the antioxidant activity, determined by measuring the decrease in the DPPH radical absorbance at 517 nm [51]. The scavenging ability exhibited by CFN samples depended on the concentration (Figure 3(a)). At 1.5 mg/mL fucoidan concentration, CFNs exhibited a scavenging abilities of 74.9% against DPPH radicals. CFNs had strong antioxidant activity due to sulfate content, and studies have indicated that these compounds, in addition to the monosaccharide content and the linear backbone regarding the polysaccharide, may promote the fucoidan bioactivity [52].  Figure 3(b) indicates that the DPPH scavenging abilities regarding CFNs were mainly derived from fucoidan. Vitamin C was treated as a positive control, and with the fucoidan concentration over 1 mg/mL, its DPPH scavenging effect was approximately 85%.

#### 3.2.2. Scavenging Ability against Superoxide Radicals

The CFNs samples exhibited dose-dependent scavenging ability for superoxide radicals. As presented in Figure 3(c), scavenging activity increased linearly within the concentration range of 0.02–0.18 mg/mL. CFNs at 0.18 mg/mL exhibited scavenging activities of approximately 50.14%, which was a comparable to the scavenging activity against DPPH that was approximately 45.73% at 0.16 mg/mL. Also, CFNs presented much stronger superoxide radical-scavenging ability relative to fucoidan alone, likely due to the ability of the amino group in chitosan to alter the compound polarity and, thus, affect the antioxidant ability. Moreover, it has been previously demonstrated that tested samples possessed much better superoxide radical scavenging ability than that against other radicals like hydroxyl and DPPH and also possessed a stronger reducing power, as the superoxide anion acts as a reduced form regarding the molecular oxygen generated by receiving one electron, which exhibits weak activity and exhibits less harmful effects on the organism and thus can be more easily scavenged relative to hydroxyl and DPPH radicals [51].

#### 3.2.3. Scavenging Ability against Hydroxyl Radicals

Figure 3(d) gives the scavenging abilities of the CFNs against hydroxyl radical inhibition, and these abilities also depended upon the dose response. The scavenging effect greatly weakened within the concentration range (1–3.5 mg/mL) of the samples. CFNs exhibited scavenging ability of approximately 35.75% at 3.5 mg/mL, indicating considerable scavenging hydroxyl ability. A previous study had investigated some antioxidant mechanisms including the inhibition regarding the generation of hydroxyl radical and cleaning hydroxyl radical [53]. The former concerns metal ion transition, as when these are lacking, H_2_O_2_ is fairly stable. Another assay system saw similar iron chelating ability and change trend to the order of the scavenging ability exhibited by hydroxyl radicals. The antioxidant activities exhibited by the examined samples exert the function by combining many factors instead of involving only a single factor.

### 3.3. Effects of CFNs on Intracellular ROS Production

The intracellular antioxidant activity was determined using the DCFH-DA assay that involves the entry of DCFH-DA into cells for forming compound with high fluorescence, i.e., dichlorofluorescein (DCF) as presented in Figure 3(e). The increased ROS levels after adding LPS and IFN-*γ* were easily visualized under fluorescence microscopy, while a reduction in intracellular fluorescent signals was observed in the CFNs groups. This suggested the effective reaction of CFNs with ROS; ultimately, the concentration could reduce. The flow cytometric analysis assisted in confirming the quantification level regarding generated ROS (Figure 3(f)). The ROS levels increased in the LPS-induced group and significantly decreased in the CFN groups.

### 3.4. Effects of CFNs on Foam Cell Formation Induced by oxLDL in Macrophages

Foam cells are major contributors to the development of AS, and oxLDL is a component of foam cells [54]. RAW 264.7 cells were exposed to ox-LDL to establish a foam cells model, and ORO was used to detect the effects of CFNs on their accumulation in RAW 264.7 cells. A large amount of oxidized lipoproteins in the model group (oxLDL-treated) was detected compared to that in control group (Figure 3(g)), indicating that the macrophages were transformed into foam cells. In contrast, the results of the CFNs addition groups indicate that there were significantly less positively stained cells than that in the model group, particularly at a dosage of 1.5 mg/mL with a reduction of approximately 59.5% (Figure 3(h)). In addition to the ROS inhibition abilities, the CFNs could also notably preserve macrophages by disrupting foam cell formation.

### 3.5. Anti-inflammatory Activities of CFNs

LPS is obviously capable of initiating a signaling cascade for inflammatory mediator expression including the cytokines involved in the innate immune responses mediated by macrophage [55]. To unravel their anti-inflammatory effects, we added CFNs at the indicated concentrations to RAW 264.7 macrophage cultures following 24 h of LPS and IFN-*γ* stimulation. It is important to find that IL-6, IL-1*β*, and TNF-*α* secretion presented an obvious increase (*P* < 0.05). Figures 3(i)–3(l) indicate how CFNs affected the pro-/anti-inflammatory cytokines secretion by RAW 264.7 macrophages stimulated by LPS. The results revealed that CFNs treatment greatly suppressed the IL-6, IL-1*β*, and TNF-*α* secretion levels in RAW 264.7 macrophage cultures stimulated by LPS (*P* < 0.05). The findings of this study suggest that CFNs may decrease the pro-/anti-inflammatory cytokine levels to exhibit anti-inflammatory potential upon macrophages stimulated by LPS.

### 3.6. Effect of CFNs on Apoptosis in RAW 264.7 Cells

**Figure 4:**
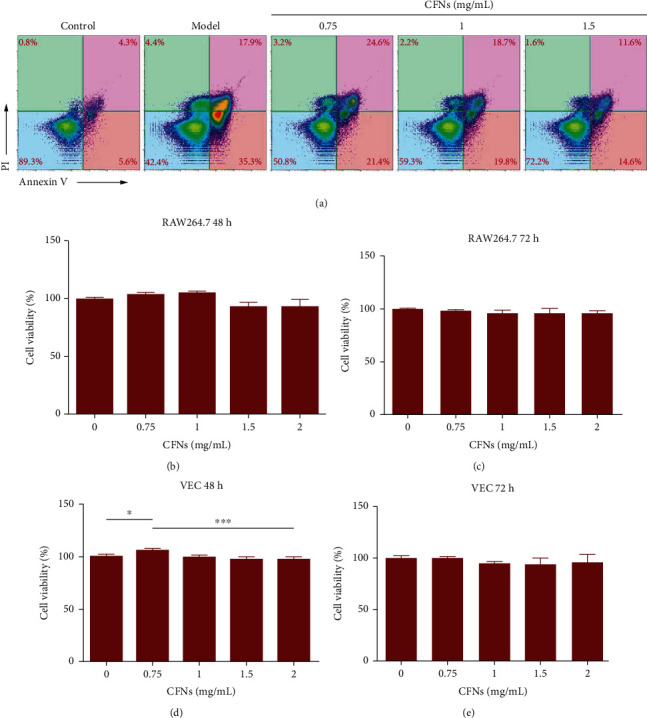
(a) Apoptosis of RAW264.7 macrophages after different treatments was determined by flow cytometry followed by Annexin V-PI double staining. (b–e) Effect of CFNs on RAW264.7 cells (b and c) and vascular endothelial cells (d and e) at different time points (48 h and 72 h). Data are means ± S.D. (*n* = 3).

The antiapoptotic effect of the CFNs was investigated using flow cytometry (Figure 4(a)). The LPS-treated group reported markedly higher percentage of TUNEL-positive cells relative to control group. In contrast, treating cells with CFNs significantly reduced the apoptotic proportion of apoptotic cells, and treating with a dose of 1.5 mg/mL exhibited the greatest effect in both early and late stage apoptosis (*P* < 0.05). These results indicate that CFNs exert antiapoptotic effects.

### 3.7. *In Vitro* Cytotoxicity Assays

The cytotoxicity of NPs typically depends upon the amount of NPs taken up by cells, and this, in turn, may depend on the concentration of the added NPs and incubation time [56]. The study focused on evaluating the cytotoxicity regarding CFNs after 48 and 72 h of incubation with the cells; during this fixed period of time, the cells were allowed to interact with different doses of CFNs. As shown in Figures 4(b)–4(e) all CFN-treated cell lines exhibited viability >92% viability after 48 h of incubation (even at 2 mg/mL of CFNs), and the percentage of viable cells was still greater than 90%, thereby indicating that cell viability was not highly affected at 48 h. At 72 h, no significant viabilities were observed for either RAW 264.7 macrophage and MVECs, indicating that CFNs exerted apparently low cytotoxic effects on cell lines and that their viability was typically not affected by CFNs despite the doses.

### 3.8. Cellular Uptake of CFNs *In Vitro*

**Figure 5:**
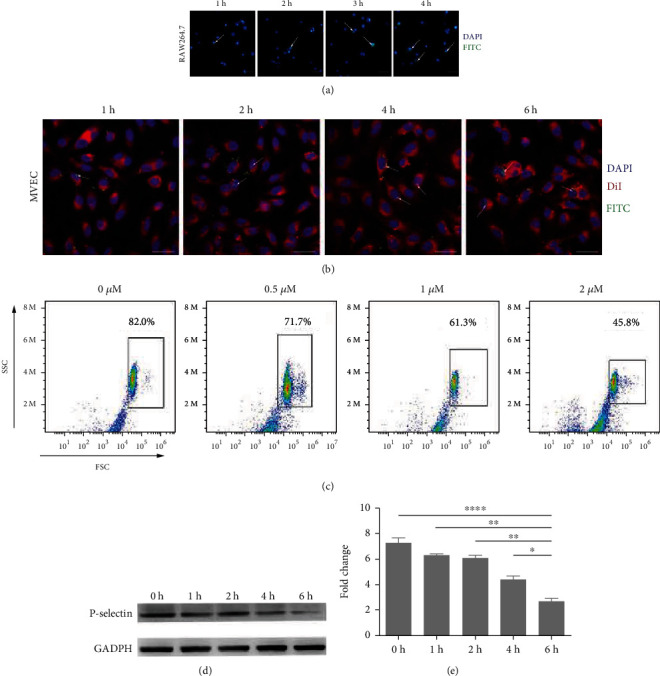
Confocal microscopy images of time-dependent cellular uptake of CFNs by (a) RAW 264.7 macrophage cells and (b) vascular endothelial cells. (c) Flow cytometry analysis of the cellular uptake of CFNs after pretreatment with P-selectin inhibitor for mouse vascular endothelial cells (MVECs). (d) Western blot analysis showing the expression of P-selectin in MVECs after incubation with CNFs. (e) Quantification of P-selectin protein on MVECs using western blot. Scale bars: (a) 500 *μ*m. (b) 50 *μ*m. Data are means ± S.D. (*n* = 3).

For the delivery of NPs into the intracellular compartments, endocytosis is usually the initial step, and the recruitment of macrophages exerts an important effect on the site-specifically delivering NPs to atherosclerotic lesions [57]. Therefore, the uptake of CFNs by macrophages RAW 264.7 and MVECs was studied by using a fluorescent microscope. Fluorescence microscopic images were obtained after treatment with CFNs for different time periods, as shown in Figure 5(a). Intracellular loading of CFNs was maximal at 4 h, a large number of intracellular fluorescent signals generated following 1 h of culture, and a further increase in the fluorescence signal was observed thereafter, indicating that macrophages can rapidly endocytose CFNs and that the uptake increased linearly with incubation time. For MVECs, the intracellular loading of CFNs was extended to 6 h, as they were not stimulated by LPS/IFN-*γ*, and the endocytosis abilities were not as strong as were those of macrophages. Similar uptake was detected after 1 h of loading, and at 6 h, the uptake increased with more fluorescence membrane (Figure 5(b)). Collectively, these findings demonstrate that CFNs are efficient vehicles that can be used to deliver drugs.

### 3.9. Targeting Ability of CFNs and P-Selectin Expression Evaluation

For confirming how P-selectin affected the CFNs cellular uptake, we adopted flow cytometry for measuring the MVECs uptake regarding the CFNs before and after adding an inhibitor of P-selectin. MVECs were first pretreated by using 0.5-2 *μ*M of P-selectin inhibitor, followed by 6 h of incubation using 1.5 mg/mL of FITC-tagged CFNs. As shown in Figure 5(c), MVECs uptake of CFNs reduced following 1 h of pretreatment with different concentrations of the P-selectin inhibitor, and the uptake decreased by ~44% after the addition of 2 *μ*M of the inhibitor, whereas it was reduced by ~12% for inhibitor concentration of 0.5 *μ*M. P-selectin expression in ECs mimicking the endothelial microenvironment in atherosclerosis was further assessed by western blotting of proteins from CFN-tagged MVECs. P-selectin expression was consistently decreased in a time-dependent manner, with the lowest protein observed at 6 h after CFNs incubation (Figures 5(d) and 5(e)), thereby demonstrating that CFNs targeting MVECs remarkably downregulated P-selectin expression levels. These results suggest that the P-selectin effectively mediates internalization of CFNs by MVECs and could provide a potential targeting site for the delivery of CFNs for use as imaging and therapeutic agents [58–60].

### 3.10. Homing to Atherosclerotic Plaques by CFNs in ApoE^−/−^ Mice

**Figure 6:**
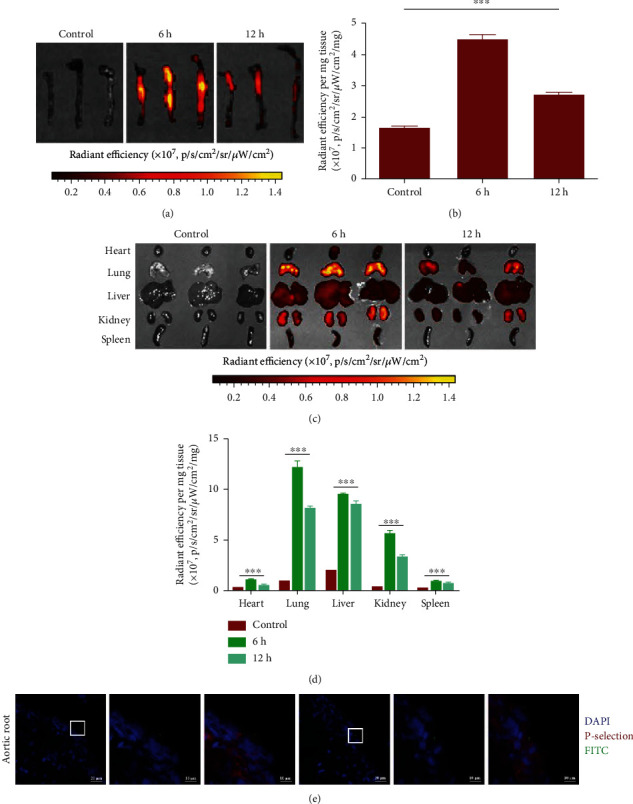
CFNs accumulation in atherosclerotic plaques of ApoE^−/−^ mice in vivo. (a) Ex vivo fluorescence imaging and (b) quantified data of the aorta after i.v. injection FITC-labeled CFNs. (c) Ex vivo fluorescence imaging and (d) quantified data of the major organs including the heart, lung, liver, kidney, and spleen after i.v. injection FITC-labeled CFNs. (e) Fluorescent microscopic images confirmed CFNs (green) targeting to atherosclerotic plaques of aortic root in ApoE^−/−^ mice. Data are means ± S.D. (*n* = 3).

For confirming if circulating CFNs could be homed to components of atherosclerotic plaques such as activated platelets *in vivo*, we explored the *in vivo* pharmacokinetic profile regarding CFNs in ApoE^−/−^ mice. At 24 h after the intravenous injection of CFNs into atherosclerotic mice, fluorescence imaging specific for CFNs was almost completely cleared from the blood. At 6 h after CFNs injection, *ex vivo* fluorescence imaging regarding the aorta displayed significant fluorescence, revealing that the preferential accumulation of CFNs within atherosclerotic plaques and their radiant efficiency was much higher relative to that from normal aortas of CFNs-treated mice. At 12 h, a less significant distribution of CFNs within the aortas was observed (Figure 6(a)), and fluorescence imaging at 6 h postinjection displayed a 1.67-fold greater radiant efficiency than that observed at 12 h postinjection (Figure 6(b)). Moreover, the accumulation of CFNs in major organs like the heart, lung, liver, kidneys, and spleen was also examined (Figures 6(c) and 6(d)), and the results indicated a similar time-dependent accumulation profile. CFNs were intravenously injected in ApoE^−/−^ mice which had established plaques, followed by the immunofluorescence analysis. Enhanced green CFNs accumulation in the atherosclerotic plaques was clearly observed. P-selectin expression *in vivo* was also clearly observed (Figure 6(e)), thereby indicating that it is an activated adhesion molecule in atherosclerosis and thrombus that allows for the attachment of CFNs [61].

### 3.11. Antiatherosclerosis Efficacy by CFNs

**Figure 7:**
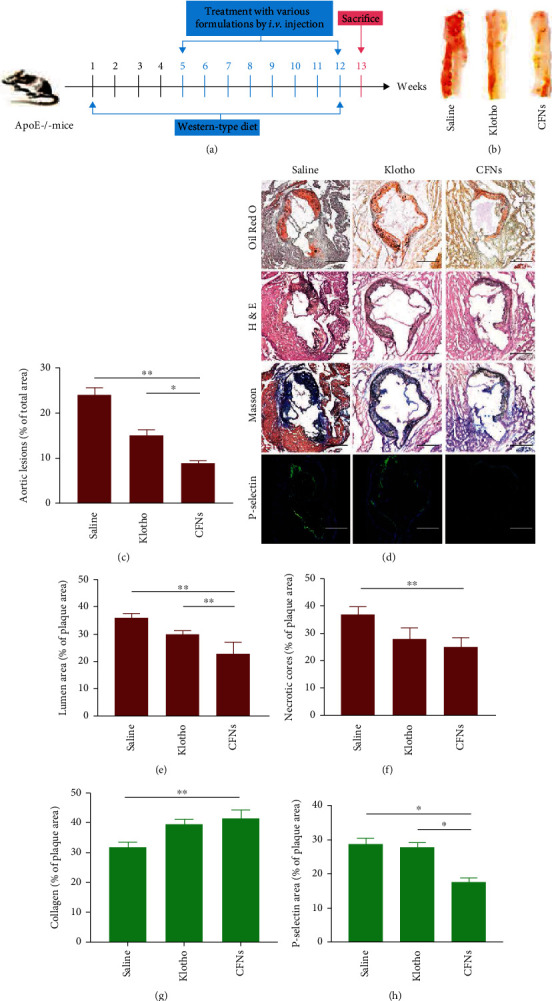
In vivo anti-atherosclerosis efficacy of CFNs. (a) Schematic diagram of the treatment protocols. (b) Representative photographs of en face ORO-stained aortas from mice after different treatments and (c) quantitative of percentage of plaque area compared with total luminal surface area. (d) Pathological and immunofluorescence detections on the sections of aortic roots from ApoE^−/−^ mice after different treatments. (e–h) Quantitative analysis on (e) lumen area, (f) necrotic cores, (g) collagen content, and (h) P-selectin. Scale bars: (c) 500 *μ*m. Data are means ± S.D. (*n* = 3).

An atherosclerotic model established in ApoE^−/−^ mice assisted in assessing the *in vivo* antiatherosclerosis efficacy. Our preliminary studies indicated that atherosclerotic plaques may come into being following 1 month of consumption of a high-fat diet and are completely formed after 3 months. Accordingly, prophylactic therapies with specific formulations were carried out through intravenous injection twice a week following 1 month of a high-fat diet (Figure 7(a)). 2 months of treatment later, the entire aorta was collected, together with ORO staining. Considerable atherosclerotic lesions were formed in the control mice treated with saline (Figure 7(b)). In contrast, the aortas remarkably reduced in areas staining by ORO in CFNs-treated mice, indicating the inhibition of the plaque development. Quantification revealed that the average plaque area exhibited a decrease in the klotho group but was obviously higher relative to the CFN group (*P* < 0.05). This indicated that the effect of CFNs was better than that of the positive control klotho groups (Figure 7(c)).

**Figure 8:**
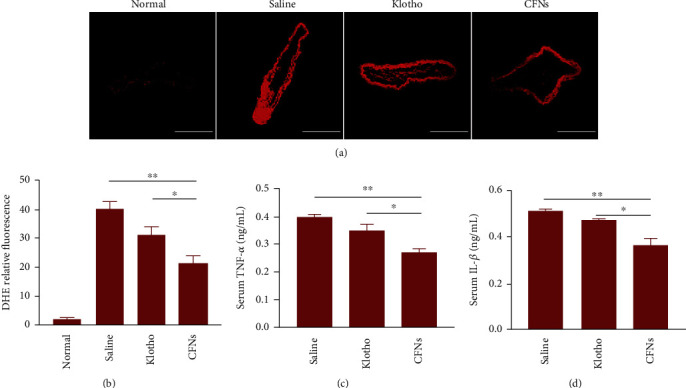
(a) Fluorescence images and (b) quantitative analysis of DHE-stained sections of brachiocephalic artery from ApoE^−/−^ mice subjected to different treatments. (c and d) Serum levels of TNF-*α* (c) and (d) IL-1*β*. Scale bars: (a) 200 *μ*m. Data are means ± S.D. (*n* = 3).

Similarly, cross-sections regarding aortic roots stained by ORO also indicated that lesions presented an obvious reduction in the CFN group relative to the other two groups. The plaque area presented a decline by 36.5% due to the CFN treatment. Consistent with this observation, H&E staining revealed that plaques in the saline group comprised acellular cores that were characteristic of complex lesions. The CFN group exhibited an obvious decrease in area of necrotic cores that ranged from 37.0 ± 2.8% to 24.9 ± 3.6% (*P* < 0.05) (Figures 7(d)–7(f)). Separate staining with the P-selectin antibody indicated that CFNs effectively adhered to P-selectin molecules, thus reducing the positive area by 39.1%. As there is a positive association between necrotic core areas and the macrophage infiltration level and plaque vulnerability, CFNs therapy is capable of effectively stabilizing atherosclerotic plaques [62]. Additionally, the Masson's trichrome method revealed enhanced fibrous cap thickness around the plaques of CFN-treated groups (Figures 7(d), 7(g), and 7(h)). These results suggest that targeted P-selectin delivery by CFNs strikingly attenuates atherosclerotic development and stabilizes the atherosclerotic plaques. Moreover, after the fluorescent probe dihydroethidium (DHE) staining, the saline-treated ApoE^−/−^ group mice displayed bright fluorescence from the superoxide anions, while the observation and quantitative analysis of the fluorescence was significantly reduced by treatment with the different formulations. The ROS level was the lowest in the CFN-treated group (*P* < 0.05) (Figures 8(a) and 8(b)). Hence, the lowest TNF-*α* and IL-1*β* expression in the serum was detected in CFN-treated group mice, indicating that CFNs therapies were more effective than the klotho treatment with regard to attenuation of systemic oxidative stress and inflammation (Figures 8(c) and 8(d)).

### 3.12. P-Selectin Expression in Peripheral Blood

**Figure 9:**
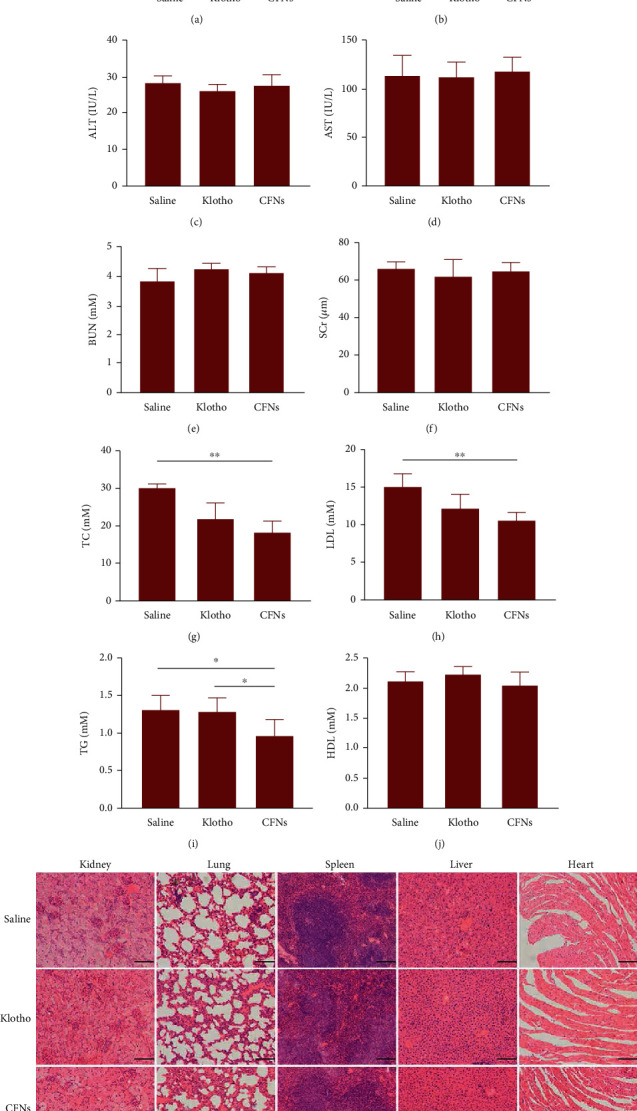
(a) Activation of P-selectin in peripheral blood of ApoE^−/−^ mice after treatment with different formulations.(b–j) Long-term safety of CFNs in ApoE^−/−^ mice after treatment with different formulations; (b) levels of hematological parameters red blood cell (RBC), (c–f) biochemical markers, and (g–j) serum lipids of ApoE^−/−^ mice after long-term various treatments. (m) Histological evaluation of organs (heart, liver, spleen, lung, and kidney.) from ApoE^−/−^ mice two months posttreatments. Scale bars: 100 *μ*m. Data are mean ± S.D. (*n* = 5).

Previous studies have demonstrated that platelet-derived P-selectin forms many steady platelet-leukocyte aggregates for promoting the atherosclerotic lesion progression and the arterial thrombogenesis [63]. Hence, flow cytometry results revealed the significant increase in the PLT activation marker P-selectin expression in the peripheral blood of ApoE^−/−^ mice in the CFNs treatment groups relative to the klotho treatment groups and saline control (*P* < 0.05). However, the klotho and saline groups presented no significant difference (*P* > 0.05) (Figure 9(a)), indicating that the CFNs attenuated atherosclerosis by targeting P-selectin and inhibiting platelet activation.

### 3.13. Long-Term Safety of CFNs in ApoE^−/−^ Mice

We investigated CFN safety following 2 months of treatment. Atherosclerosis is a persistent disease involving many factors and is typically treated as a lipid accumulation-induced chronic inflammation form. Therefore, we evaluated the lipid profiles regarding mouse models after CFNs treatment. The TRIG, CHOL, and LDL levels in the control group were markedly elevated, indicating that the ApoE^−/−^ mice experienced a lipid disorder after 3 months of western diet. Compared to the control groups, at the end of 3 months, for both the CFNs and klotho groups, plasma TC, LDL, and TG levels presented an obvious decrease, indicating that CFNs had better protective effects to those of klotho with regard to inhibition of lipid accumulation (Figures 9(g)–9(j)). Complete blood count results found no obvious difference among various groups in terms of the RBC (Figure 9(b)). Additionally, ALT, AST, BUN, and SCr levels were not different among the groups, indicating that CFN treatment failed to impact the hepatic or kidney functions (Figures 9(c)–9(f)). The potential damaging effects of CFNs on highly vascularized organs like the heart, liver, lung, spleen, and kidney were also examined. Two months posttreatment, examination regarding the H&E-stained sections of the tissues from the treatment groups revealed no apparent differences in morphological structures when compared to the saline group. This indicates that CFNs may not cause significant adverse effects that are relevant to atherosclerosis (Figure 9(k)).

## 4. Discussion

P-selectin (CD62P) belongs to the selectin family regarding cell adhesion molecules and takes charge of responding to vascular injury in platelets and endothelial cells, as well as VSMCs. Facing the development of thrombosis develops and the occurrence of inflammation, P-selectin is capable of quickly transferring to platelets and endothelial cell surfaces [64, 65]. Both endothelial and platelet P-selectin also contribute to the maturation of atherosclerotic lesions, and previous studies have demonstrated that lesions where both cell types express P-selectin exhibit a tendency to be more mature than are those where no P-selectin is expressed [61], suggesting that P-selectin presents an attractive potential therapeutic target for atherosclerosis therapies. Fucoidan is a kind of natural sulfated polysaccharide from brown seaweeds. Fucoidan presents beneficial effects on biology; hence, it is highly valued as an ingredient promoting the functions of food, health products, and pharmaceuticals [66, 67]. Fucoidan of which a molecular weight is about 7.2 kDa acts as an excellent ligand for P-selectin [39], indicating that it can serve for the preparation of CFNs with certain targeting against atherosclerotic plaques.

In this study, a preliminary investigation was performed to verify the different parameters of polyelectrolyte complexation to optimize the functional properties of the formed CFNs. Our results revealed similar CFNs in size with various polymer concentrations and higher PDI values for both the high and low polymer concentrations. As marine polysaccharides, fucoidan and chitosan are weak polyelectrolytes, and the solution pH value decided the electrostatic interactions' degrees. It is established that the amino groups' pKa value on CS is about 6.5, and when the pH value increases, amino groups tend to be deprotonated and CS would be precipitates [18]. The number of positive charges in solution is insufficient to form a complex with negative charges, thereby leading to the formation of unstable NPs [68]. The results can be explained based on CS-to-fucoidan weight ratio. In the study, we tested the pH response of CFNs with different CS-to-fucoidan weight ratios that included 1:1 (1C/1F), 2 : 1 (2C/1F), and 3 : 1 (3C/1F), and our results indicated considerable swelling of 1F/1C NPs as the pH level increased. The particle size became unmeasurable when the pH reached approximately 6.0 (data not shown); therefore, these particles presented a considerable response to pH variations. In contrast, the 2F/1C and 3F/1C NPs were almost unchanged as the pH increased from 2.5 to 6.0, and 3F/1C NPs swelled moderately but remained spherical in shape at pH levels of 7.4 (corresponding to the pH of blood). Additionally, the 3F/1C NPs exhibited a low transmittance, suggesting that the CFNs were stable and comparably insensitive to pH changes. These effects were related to the deprotonation of amine groups on CS; CFNs with a higher weight ratio of CS-to-fucoidan carry more positive charge on their surfaces and may adhere to the intravascular wall of blood vessels and then destabilize and disintegrate due to the pH gradient on the basolateral sides of the plaque macrophage area or epithelial cells, and this is suitable for drug delivery and topical administration [69, 70]. We also tested the 4 : 1 and 5 : 1 CS-to-fucoidan weight ratios, but the CFNs presented a higher PDI that was suggestive of a less controlled process. Therefore, we finally selected the 3 : 1 ratio for the experiments, and the concentrations of chitosan were varied according to those of fucoidan. Considering the further application of CFNs under physiological conditions, we tested a cross-linking agent (EDC/NHS) to enhance their stability and control the release of antioxidants. EDC is used to crosslink amines (NH_2_) to carboxylate groups, and NHS enhances the activity by activating the carboxylate group [71, 72]. The basic principle in this case is to crosslink the amino group of CS to the carboxyl group of uronic acid in the fucoidan structure [64]. We evaluated various concentrations of EDC/NHS and set 120 mM NHS+300 mM EDC to optimize the CFN size and PDI. A previous study reported that oral administration of fucoidan and other marine polysaccharides are the promising applications [73]; however, in literature, the possibility of CFNs achieving resistance to pH variations in human organisms is not available. The pH characteristics of human organisms vary according to the physiology of each organ or tissue (pH 6-7 in the duodenum, jejunum, and ileum; pH 1-3 in the stomach; and pH 7.4 in the body fluid underneath the epithelial cells) [74]. In this study, we administrated the CFNs intravenously to mice to ensure a better effect on plaques. However, further investigations are required to evaluate the optimal production parameters of CFNs such as polymer concentration and ratios and pH of the solution that could allow for a promising characterization of CFNs for oral administration in both the pharmaceutical and functional food industries.

After optimization, the antioxidant activities of the developed CFNs were investigated using a DPPH scavenging assay. DPPH, a stable radical, widely serves for evaluating antioxidant activities over a relatively short period of time, and compared to other antioxidants, it was capable of determining the antiradical power exhibited by the developed CFNs via the analyses that incorporated DPPH. The scavenging effect principle is related to the hydrogen donating ability exhibited by the antioxidant. When the DPPH free radical encounters a proton-donating substance, it forms a stable DPPH-H molecule, and the reduction of DPPH to DPPH-H could be measured at an absorbance of 517 nm [31, 50]. As shown in Figure 3(a), the inhibition percentage of CFNs was concentration-dependent, and CFNs obtained using fucoidan at 1.5 mg/mL demonstrated DPPH scavenging activity of 74.9%. The DPPH scavenging activity was primarily attributed to fucoidan, but it exhibited a remarkably lower inhibition percentage relative to the positive control group (vitamin C) over the same range of concentrations (Figure 3(b)). Additionally, the scavenging abilities of CFNs against superoxide and hydroxyl radicals were dose dependent. The superoxide anion radical is the first ROS generated by the biological and photochemical reactions [75]. Despite being remarkably weak, it is capable of indirectly initiating lipid peroxidation by means of decomposing to other stronger ROS such as singlet oxygen and hydroxyl radicals [76]. On that account, superoxide scavenging can greatly impact antioxidant activity. In this study, the PMS–NADH superoxide generating system served for assessing superoxide radicals, and the absorbance at 560 nm presented an increase with the superoxide anion being scavenged by CFNs, indicating the high antioxidant activity regarding the sample. The hydroxyl radical is an oxidant with high efficiency, capable of reacting with a majority of biomacromolecules functioning in living cells as well as triggering serious damage to biomolecules nearby [77]. Hence, the removal of hydroxyl radicals critically helped to improve antioxidant defenses in cell systems.

Overproduced ROS are considered to trigger tissue and cell inflammation that activates a network of inflammatory signals and in turn increases acute-phase reactants and other mediators [78]. The mechanism may involve several intracellular signaling pathways, and it has been demonstrated that LPS/IFN-*γ* and the elevated ROS levels stimulated mitogen-activated protein kinase (MAPK) and NF-*κ*B transcription factors [79, 80]. MAPK pathway signals transduce into various cellular processes, including cell differentiation, proliferation, and apoptosis [81], that subsequently enhances the release of inflammatory cytokines like TNF-*α* and IL-1*β* [82, 83]; NF-*κ*B is required for transcription of iNOS gene in some cell lines and can mediate in a stepwise manner the oxidation of L-arginine to form NO [84]. Here, we examined if CFNs could inhibit intracellular ROS production and inflammatory cytokines in RAW 264.7 macrophages induced by LPS/IFN-*γ*. On probing with DCFH-DA, the increase in ROS after LPS/IFN-*γ* stimulation was substantial in the model group (Figure 3(e)). In contrast, the increased ROS levels in all CFN groups were comparably weakened, particularly at a fucoidan concentration of 1.5 mg/mL. This was determined by flow cytometric analysis that revealed a significant reduction in the CFN groups. Additionally, studies have demonstrated a direct correlation between oxLDL uptake and generation of ROS by these cells [85], and the scavenging of intracellular ROS by NPs results in their inhibition of oxLDL receptor expression [54]. Moreover, oxLDL triggers the transformation of macrophages into lipid-laden foam cells that critically affected atherosclerosis' occurrence and progression [86]. Subsequently, we further examined if CFNs were effective with regard to inhibition of macrophage formation in foam cells. The results were in accordance with the ROS inhibition testing; CFNs decreased oxLDL uptake by macrophages in a concentration-dependent manner with the lowest level of foam cell formation at the greatest CFNs testing dosage, indicating they could effectively reduce macrophage internalization regarding oxLDL as well as attenuate the formation regarding foam cell. In the anti-inflammatory assay, due to LPS treatment, proinflammatory cytokines (TNF-*α*, IL-1*β*, and IL-6) and NO increased significantly in RAW 264.7 macrophages, and the responses were attenuated in the CFN groups. However, contrary to the results obtained using a concentration of 1.5 mg/mL group, a dosage of 0.75 mg/mL did not have any significant effects in the model groups with regard to all proinflammatory cytokines and NO testing; moreover, a dosage of 1 mg/mL only exhibited limited effects with significant differences in TNF-*α* and IL-1*β* testing, and previous findings revealed that a sulfated polysaccharide derived from the brown seaweed *Fucus vesiculosus* acted by through suppressing the NF-*κ*B activation and downregulating the MAPKs pathways [87]. Our results substantiated observation that the CFNs displayed concentration-dependent attenuation effects that may be largely due to fucoidan characteristics (containing sulfated polysaccharides). We used the high dosage groups for further study due to their stable and inflammation-modulatory abilities.

The NPs' cytotoxicity depends upon the amount of NPs taken up by cells, and this is affected by the concentration of NPs that are incubated with the cells [56, 88]. When CFNs were subject to a cytocompatibility test, a slight reduction in the viabilities of RAW 264.7 macrophages after 48 h of culture was observed without significant differences at dosages of 1.5 and 2 mg/mL. However, at a dosage of 0.75 mg/mL, relatively high cell viabilities of 103.9% and 105.7% were detected for both RAW 264.7 and endothelial cells, respectively. At 72 h, the cell viabilities were not typically affected by the various doses of CFNs. Although fucoidan NPs can induce cell apoptosis and lead to a decline in cell viability, this apoptosis-inducing activity primarily results from the anticancer effect of fucoidan [42]. For nontumor cells such as RAW 264.7 cells, fucoidan NPs were inclined to protect them from ROS injury due to their antioxidant activities [38]. Thus, the decline in viability regarding RAW 264.7 macrophages after treatment by CFNs for 48 h was likely due to necrosis of cells or to their natural process of apoptosis and not from the effects of CFNs. This is in agreement with our apoptosis detection revealing protective effects against H_2_O_2_-induced injury in RAW 264.7 macrophages in a concentration-dependent manner. The cationic polymers' cytotoxicity is directly related to their surface charge density [38]; however, chitosan at concentrations as high as 10 mg/mL has not been observed to induce substantial histological changes in absorptive cells [89]. It is valuable to note that it possesses little cytotoxicity regardless of its anionic surface charge. Hence, in subsequent cellular uptake studies, a fucoidan concentration of 1.5 mg/mL in CFNs was selected, as the cells were not influenced by toxic effects in the experiment. Fucoidan has been reported to bind to macrophage surface scavenger receptors and type A I and II transmembrane glycoprotein receptors [90, 91], while chitosan interacts with cell membranes by nonspecific electrostatic forces of attraction without specific receptors [92, 93]. Although macrophages in the atherosclerotic plaques are located in an inflammatory microenvironment that are full of proinflammatory mediators, we investigated whether their phagocytic capability of CFNs are affected by the inflammatory stimulation [34]. It was found that internalization of CFNs increased significantly at various time points (Figure 5(a)) after the 24 h of prestimulation of macrophages with IFN-*γ* and LPS. Furthermore, the CFNs agglutinated to a high degree around cells in the cellular uptake studies, with macrophages, in particular, exhibiting large amounts of agglomeration. For confirming how P-selection affected the cellular uptake regarding CFNs, flow cytometry assisted in measuring the MVECs uptake regarding CFNs prior to and after adding an inhibitor of P-selection. Our results supported our hypothesis regarding internalization of the CFNs mediated by P-selection, and the lowest protein expression of P-selectin was achieved at 6 h of CFNs targeting.

In this study, we introduced the cardiovascular disease-related suppressor protein klotho as a positive control. Klotho was originally identified as an antiaging protein with high expression in the kidneys [94]; it has been proven that the extracellular domain regarding klotho protein is capable of exerting physiological effects as a circulating hormone, e.g., helping endothelial cells to avoid oxidative injury and dysfunction related to atherosclerosis [95]. However, emerging evidence from clinical trials has revealed that klotho therapies may disrupt Ca^2+^ homeostasis by controlling parathyroid hormone (PTH) secretion, and intravenous infusion of klotho may also cause hypophosphatemia and phosphaturia [96–98]. Hence, such limitation may be addressed by replacing klotho with NPs as they can precisely target towards the adhesion molecules in atherosclerotic lesions with minimal side effects on the endocrine system. Previous studies indicated that free klotho at a concentration of 20 *μ*g/kg body may produce maximal endothelium protecting effects [43]; in this sense, in the vivo studies, 0.4 *μ*g/mL klotho was the chosen concentration as the positive control group. Before evaluating the therapeutic efficacy of CFNs, we first investigated the atherosclerotic plaque targeting capability regarding CFNs. After intravenously injecting CFNs into ApoE^−/−^ mice, the relative fluorescence intensity of the aorta at after 6 h administration was approximately 1.67-fold higher than that at 12 h after administration (Figure 6(b)), which indicates that aggregation of CFNs in the aorta occurred primarily 12 h. We further investigated the accumulation of CFN in the lung, heart, spleen, liver, and kidney and observed that lung accumulation was typically the highest at 6 h while the fluorescence intensity weakened considerably at 12 h (Figure 6(c)), in a manner that was consistent with observations in the aorta. Although the liver reached its highest concentration at 12 h with and gradually decreased from 6 h, we expected it to be a clearance organ with delayed administration [99]. As the distribution in these organs was nonspecific, further immunofluorescence analysis was performed to detect the specific target in the aortic arch regions; that are prone to developing atherosclerotic plaques due to disturbed blood flow [47, 100]. We observed the presence of gathered CFNs taken up by atherosclerotic plaques. The adhesion of CFNs to the plaques mediated by P-selectin was measured by immunofluorescence, and the fluorescent imaging clearly detected the CFNs that adhered to either lesional platelets or endothelial cells.

Consistent with the *in vitro* study, the antiatherosclerosis targeted effect of CFNs *in vivo* was assessed by immunofluorescence examination of P-selectin in the aortic root section. The results revealed a decline in the fluorescence intensity of marked P-selectin in the atherosclerotic plaque area after treatment with CFNs, but not by klotho, as the treatment effects of the latter were not dependent on the mediation of P-selectin. The process was corroborated by the decreased activity and expression of serum proinflammatory cytokines (IL-1*β* and TNF-*α*), where the CFN-treated group exhibited lower levels than that of the klotho-treated group due to CFN mediated blocking of leukocyte recruitment and rolling on platelets and endothelium. In agreement with this result, for the cross-sections of aortic roots stained with ORO, HE, Masson's trichrome, and dihydroethidium (DHE), despite the variability of animal model, the CFN-treated group was more efficient in reducing both systematic and local inflammation lesions and oxidative stress compared to treatment by free klotho. Studies have demonstrated that platelet activation is a triggering factor for atherosclerosis as well as inflammation [101], and a high blood platelet count is an independent contributor to venous thrombosis [102]. P-selectin can be expressed rapidly on the PLTs' surface via effect of membrane fusion when PLTs are activated [46]. Therefore, detection of platelet-derived P-selectin in peripheral blood can be used as a marker for atherosclerotic lesion development. In the present study, we found that the P-selectin's expression in the CFNs treatment group was lower than that in the klotho treatment group, which was not significantly different from that in the saline group. This suggests that platelet activation is inhibited by CFNs that bind to P-selectin to block leukocyte recruitment and rolling on platelets and the endothelium. To assess biosafety, adverse effects were investigated after treatment for 4 weeks, and no distinguishable changes were observed in the major organs, indicating no injury to the major organs of ApoE^−/−^ mice. The western diet induced marked lipidemia in mice with high levels of lipid profiles, while the CFNs treatment groups displayed lower levels of TC, LDL, and TGs (Figures 9(g)–9(i)) with no significant changes observed in HDL (Figure 9(j)). We expected that CFNs may improve the levels of serum lipid by regulating cholesterol and TG synthesis enzymes in the liver [103]. Meanwhile biochemical indices of liver and kidney function (ALT, AST, BUN, and SCr) revealed no significant differences among these groups, indicating that they were not affected by the treatments. Moreover, complete blood examination implied that RBC displayed no significant variation among the various groups. Consequently, the nanoplatform used in this study did not induce significant adverse side effects or *in vivo* toxicity following intravenous administration, indicating that it is a safe candidate for the treatment of atherosclerosis.

## 5. Conclusion

In this study, marine-origin polymeric NPs with ROS-scavenging characteristics were successfully synthesized via the direct electrostatic interaction of fucoidan and chitosan. The physicochemical properties of CFNs including their size, zeta potential, and stability were decided by the chitosan/fucoidan ratio. The CFNs exhibited effective scavenging abilities against DPPH, superoxide radicals, and hydroxyl radicals. As per ROS determination tests, CFNs can also be capable of effectively inhibiting the inflammatory response as well as cell apoptosis induced by ROS in macrophages. The CFNs covalently conjugated to FITC yielded fluorescent chitosan molecules that could be formulated into labeled NPs and exerted no cytotoxic effects on macrophages and VECs. Fluorescent imaging together with flow cytometry analysis assisted in validating the P-selectin targeting potential, meanwhile improving CFN cellular uptake with MVECs. CFNs also demonstrated P-selectin-dependent atherosclerotic plaque-targeting abilities *in vivo*. The desirable therapeutic effects of CFNs were recognized by attenuating systemic oxidative stress and inflammatory cell infiltration in plaques when compared to the traditional atherosclerosis suppressor protein klotho. Importantly, anti-PLT activation by CFNs can remarkably suppress atherosclerosis. Thus, our results indicate that CFNs can served as a promising antiatherosclerotic nanotherapy, which shall be further studied.

## Figures and Tables

**Table 1:** Nanoparticles characterization by dynamic light scattering.

Technique	Property	Value
DLS	Size (nm)	151.9 ± 5.8
	PDI	0.192 ± 0.012
	Zeta potential (mV)	25.1 ± 0.36

## Data Availability

The data used to support the findings of the present study are available from the corresponding authors upon request.

## Abbreviations

ALT: Alanine aminotransferase
ALB: Albumin
AST: Aspartate aminotransferase
BUN: Blood urea nitrogen
BSA: Bovine serum albumin
CFNs: Chitosan–fucoidan nanoparticles
CK: Creatine kinase
DAPI: 4′,6-Diamidino-2- phenylindole
DCF: Dichlorofluorescein
DiO: 3,3′-Dioctadecyloxacarbocyanine perchlorate
DHE: Dihydroethidium
DLS: Dynamic light scattering
DMEM: Dulbecco's modified Eagle's medium
DMSO: Dimethyl sulfoxide
DPPH: 1,1-Diphenyl-2-picrylhydrazyl
ELISA: Enzyme-linked immunosorbent assay
EDTA: Ethylenediaminetetraacetic acid
FITC: Fluorescein isothiocyanate
FTIR: Fourier transfer infrared spectroscopy
H&E: Hematoxylin and eosin
HDL: High-density lipoprotein
LDH: Lactate dehydrogenase
LDL: Low-density lipoprotein
LPS: Lipopolysaccharide
NADH: Nicotinamide adenine dinucleotide-reduced
NBT: Nitro blue tetrazolium
NGS: Normal goat serum
ORO: Oil Red O
oxLDL: Oxidized low-density lipoprotein
PDI: Polydispersity index
PWL: Paw withdrawal latency
PBS: Phosphate-buffered saline
PEI: Polyethylenimine
PLGA: Poly(lactic-co-glycolic acid)
PLL: Poly(L-lysine)
PMS: Phenazine methosulfate
RBC: Red blood cell
ROS: Reactive oxygen species
SCr: Serum creatinine
SD: Standard deviation
SME: Scanning electron microscopy
TBIL: Total bilirubin
TC: Total cholesterol
TEM: Transmission electron microscopy
TG: Triglyceride
WBC: White blood cell.
